# GWAS and eQTL analysis identifies a SNP associated with both residual feed intake and *GFRA2* expression in beef cattle

**DOI:** 10.1038/s41598-018-32374-6

**Published:** 2018-09-24

**Authors:** Marc G. Higgins, Claire Fitzsimons, Matthew C. McClure, Clare McKenna, Stephen Conroy, David A. Kenny, Mark McGee, Sinéad M. Waters, Derek W. Morris

**Affiliations:** 1 Discipline of Biochemistry, National University of Ireland, Galway, Ireland; 2 Animal and Bioscience Research Department, Animal & Grassland Research and Innovation Centre, Teagasc, Grange, Dunsany, Co. Meath Ireland; 3 Livestock Systems Research Department, Animal & Grassland Research and Innovation Centre, Teagasc, Grange, Dunsany, Co. Meath Ireland; 4 Irish Cattle Breeding Federation, Highfield House, Bandon, Co. Cork Ireland; 5 Present Address: Department of Agriculture, Fisheries and the Marine, Celbridge, Co. Kildare Ireland; 6 Present Address: ABS-Global, DeForest, WI USA

## Abstract

Residual feed intake (RFI), a measure of feed efficiency, is an important economic and environmental trait in beef production. Selection of low RFI (feed efficient) cattle could maintain levels of production, while decreasing feed costs and methane emissions. However, RFI is a difficult and expensive trait to measure. Identification of single nucleotide polymorphisms (SNPs) associated with RFI may enable rapid, cost effective genomic selection of feed efficient cattle. Genome-wide association studies (GWAS) were conducted in multiple breeds followed by meta-analysis to identify genetic variants associated with RFI and component traits (average daily gain (ADG) and feed intake (FI)) in Irish beef cattle (n = 1492). Expression quantitative trait loci (eQTL) analysis was conducted to identify functional effects of GWAS-identified variants. Twenty-four SNPs were associated (*P* < 5 × 10^−5^) with RFI, ADG or FI. The variant rs43555985 exhibited strongest association for RFI (*P* = 8.28E-06). An eQTL was identified between this variant and *GFRA2* (*P* = 0.0038) where the allele negatively correlated with RFI was associated with increased *GFRA2* expression in liver. *GFRA2* influences basal metabolic rates, suggesting a mechanism by which genetic variation may contribute to RFI. This study identified SNPs that may be useful both for genomic selection of RFI and for understanding the biology of feed efficiency.

## Introduction

Feed can account for more than 75% of variable costs of beef enterprises^1^. Consequently, selection of cattle that efficiently convert feed to carcass growth would improve farm profits due to reducing expenditure on feed while maintaining protein output^2^. Moreover, there is pressure on the agricultural industry to reduce methane emissions and improve its environmental footprint, while simultaneously increasing beef output to meet the growing demand for protein worldwide^3^. Selection for feed efficient cattle could increase beef output while concurrently decreasing methane production, as it has been suggested that low residual feed intake (RFI) (feed efficient) animals emit less methane than their high RFI counterparts^4^.

RFI is a measure of feed efficiency, defined as the difference between actual and predicted feed intake (FI)^5^. RFI has been shown to be moderately heritable, with an estimated heritability of 0.33^2,6^, making it an ideal trait for selection as any genetic gain will be maintained and propagated through the cattle herd^6^. However, calculation of RFI is currently impeded by both the expense and logistics associated with its measurement, involving recording of both FI and body weight gain for each individual animal for a period of 70 days^7^. Identification of genetic markers for RFI and component traits, such as FI and average daily gain (ADG), and their incorporation into genomic assisted breeding programmes would enable more rapid and cost effective selection of feed efficient cattle^8^. Indeed, RFI has been incorporated into the Australian dairy industry’s genomic breeding programme^9^. Unlike the situation that predominates for dairy production systems worldwide, effective identification of selection markers of RFI for beef cattle must take into account a multiplicity of breeds and the mainly crossbred nature of cattle typically utilised within the global beef industry through employing multi-breed populations in order to identify variants of interest^10,11^. Differences in linkage disequilibrium (LD) between breeds may impact the association of markers and quantitative trait loci across breeds. The use of multiple breeds in a reference population is important to account for this variation in LD between breeds^10^.

In Ireland, the genomic assisted breeding programme is administered by the Irish Cattle Breeding Federation (ICBF)^12^. Markers for selection are included on the International Dairy and Beef (IDB) custom genotyping chip, which is based on the Illumina BovineSNP50 genotyping chip providing the IDB chip with genome-wide coverage^13,14^. As well as single nucleotide polymorphisms (SNPs) that are used for the genomic selection programme, the IDB chip contains SNPs for parentage verification and SNPs included for research purposes only^13^. This includes a selected subset of SNPs associated with RFI in cattle populations outside of Ireland^15–21^ which have been added to the IDB chip for research purposes to validate their use as biomarkers of RFI and associated traits in Irish beef cattle (n = 102, Supplementary Table S1).

Genetic markers for RFI can be identified via genome-wide association studies (GWAS). Several GWAS have identified SNPs associated with feed efficiency-related traits in cattle populations, both purebred and crossbred, from North America, South America and Australia^16,20–23^. Despite considerable interest in identifying markers for RFI, ADG and FI, no published GWAS has been carried out to test for associations between SNPs and these traits in Irish beef cattle. In Ireland, commercial beef cattle are mainly crossbreds, with Charolais (CH), Limousin (LM), Aberdeen Angus (AA), Belgian Blue (BB) and Simmental (SI) breeds predominating genetically^12^. This breed heterogeneity coupled with the challenges in obtaining sufficient numbers of RFI phenotypes for GWAS are primary reasons for the difficulty in applying GWAS on a large-scale basis for RFI to beef cattle in Ireland and most other beef producing nations.

It is important to identify genetic variants that underlie phenotypic variation. One method to identify SNPs that are implicated in observed variation is by carrying out expression quantitative trait loci (eQTL) studies. eQTL analysis enables investigation of the effect of genotype on gene-expression levels which may in turn affect phenotype^24^. Previous eQTL analysis carried out in mammary tissue of dairy cattle has identified several eQTLs for milk production traits enabling the identification of genes such as *PLAG1* and *MGST1* as potentially functional in the development of divergent milk production traits^25,26^. eQTLs have been identified for temperament in the adrenal cortex of crossbred German cattle^27^. Despite the ability of eQTL analysis to identify potentially causative genes for complex traits, to the best of the authors’ knowledge no eQTL analysis has been carried out for RFI in beef cattle, or in any other livestock species. Liver and muscle are key tissues of interest with regards to feed efficiency as they are both large, metabolically active tissues accounting for approximately 24% and 25% of basal energy expenditure, respectively^28,29^. Thus, investigation into the presence of eQTLs in these tissues may aid in unravelling the biology underlying divergence in feed efficiency.

Due to multiple breeds of cattle present in beef production systems, it is important to identify markers for traits that have effects across multiple breeds^10^. Thus, the objectives of this study were to: (i) perform GWAS for RFI, and its component traits, namely FI and ADG, in different breeds of Irish beef cattle and combine results in order to identify associated SNPs in a mixed breed cohort, (ii) validate a selection of internationally identified markers of RFI present on the IDBv3 chip for utility as selection markers for RFI in Irish cattle and (iii) to investigate the effects of associated variants on gene expression in metabolically important tissues using eQTL analysis, in order to understand the biological mechanisms underlying divergence in RFI and component traits.

## Methods

All biological sampling and procedures involving animals within this study were reviewed by the Teagasc Animal Ethics Committee and/or the UCD Animal Research Ethics Committee. All procedures carried out prior to 2013 were licenced by the Irish Department of Health, all procedures carried out since 2013 were licenced by the Irish Health Products Regulatory Authority in accordance with the cruelty to Animals Act 1876 and the European Communities (Amendment of Cruelty to Animals Act 1876) Regulations 2002 and 2005.

### Phenotypic data collation

Data were collated for this study from growing bulls (n = 1823), steers (n = 459) and heifers (n = 164), which had previously undergone phenotypic measurement testing in Ireland between 2006 and 2017. The average ages and standard deviations for cattle included in the phenotypic data file were available were available on a group-by-group basis (Supplementary Table S2). Throughout each phenotypic measurement trial, the health of the animals was monitored. Any animal that required treatment was noted and excluded from further analysis. Phenotypes were gathered at the national beef research centre in Teagasc Grange; UCD Lyons Research Farm, University College Dublin and the ICBF national beef performance test station, Tully, Co. Kildare, Ireland. Phenotypes were collected from both purebred and crossbred beef cattle. For crossbred animals to be included in the phenotypic dataset the proportion of genetic material from a single parental breed had to be greater than 50%. Prior to further analysis cattle were grouped by breed. Predominant breeds were LM, CH, SI, BB and AA (n = 737, 499, 413, 191 and 174, respectively), other breeds were represented at smaller numbers. The RFI range for LM, CH, SI, BB and AA was 2.69 to −2.52, 2.70 to −2.48, 2.75 to −2.82, 1.63 to −2.00, and 2.87 to −2.64 respectively.

This resulted in the generation of a phenotypic file consisting of 2,446 cattle. For 429 of these animals, data relating to breed, diet, and methods used to calculate RFI, ADG and FI has been previously described^4,28,30–33^. Records for remaining cattle were made available by the ICBF from the national beef performance test centre at Tully, Co. Kildare, Ireland.

The management protocol of the ICBF animals is described briefly here. Animals were housed in pens for the duration of their test period which was between 70 and 105 days. A Calan gate system (American Calan, Northwood, NH, USA) was used to record individual animal FI. Bulls were individually offered *ad libitum* concentrates and 3 kg fresh weight of hay, while steers were offered 8 kg concentrates and 5 kg fresh weight of hay. Hay was offered in order to maintain healthy rumen function and to reflect an Irish commercial high concentrate based dietary regimen. Refused feed was weighed weekly and subtracted from total feed offered in order to calculate total feed consumed. Dry matter intake was then calculated in order to determine FI, which was used for calculation of RFI. Cattle were weighed at the beginning and end of the test period, and every 21 days during the test period. ADG was calculated as the coefficient of linear regression of body weight on time, computed in the software package R^34^. Mid-test metabolic bodyweight (body weight^0.75^, MBW) was calculated as body weight^0.75^ in the middle of the RFI measurement period, which was estimated from the intercept and slope of the regression line after fitting a linear regression through all MBW observations. RFI was calculated for each animal as the difference between actual and predicted FI. Predicted FI for each animal was computed by regressing FI on MBW and ADG. Calculation of predicted FI was calculated for each contemporary group individually.

### Genotyping

DNA was isolated for genotyping from one of two tissue types sourced from 429 cattle described previously. Muscle was used when blood was unavailable. Blood samples were obtained by jugular venipuncture at the end of the RFI measurement period, as per Fitzsimons *et al*.^4^, and stored at −80 °C prior to use^4^. Muscle samples were obtained via biopsy of the *M*. *longissimus dorsi* following the RFI measurement period, as per Kelly *et al*.^35^, and stored at −80 °C before DNA extraction. DNA from blood samples was extracted using the Maxwell 16 Blood DNA kit (Promega, Madison, WI, USA) as per manufacturer’s instructions. DNA was extracted from muscle samples using a phenol-chloroform extraction method. Briefly, 0.1 g of frozen muscle tissue was immersed in 1 mL of Trizol and homogenized using a Precellys 24 homogeniser. 200 µl chloroform was added to the homogenate, which was then centrifuged at room temperature for five minutes at 16,000 g. After centrifugation the aqueous phase was transferred to a new tube. Two volumes of ice cold ethanol were added to the aqueous phase and this mixture was centrifuged at 16,000 g for 15 minutes at 4 °C resulting in the formation of a DNA pellet. The supernatant was removed and the pellet was washed by the addition of 1 ml 70% ethanol and centrifuged at 16,000 g for 5 minutes at 4 °C. Washing was carried out twice. Following washing, any remaining supernatant was removed and the pellet was left to air-dry. The DNA was then re-suspended in 150 µl RNase/Dnase free H_2_O.

Once DNA was isolated, samples were analysed for quality and quantity using a Nanodrop spectrophotometer. DNA of sufficient quality for genotyping was available for 422 samples. All DNA samples were normalised to a concentration of 50 ng/µl for genotyping analysis. Genotyping was carried out on the IDBv3 chip^13^ by Weatherby’s Scientific Ltd. (Johnstown, Naas, Co. Kildare, Ireland). The ICBF provided a further genotypes for 1,262 cattle that had been genotyped on the IDBv3 chip by Weatherby’s Scientific.

In addition to the 1,684 animals genotyped directly on the IDBv3 chip, 338 cattle were genotyped on the Illumina Bovine HD genotyping chip. These 338 cattle were imputed to IDBv3 density using Fimpute version 2.2^36^. The reference population used for Fimpute was 50,000 Irish cattle with genotyped parents. Imputation of all 338 cattle was conducted across breed type to reflect the Irish national cattle population.

Once genotyping and imputation were complete the study consisted of 2,022 animals with genotypic data for all IDBv3 markers. This genetic data was uploaded to the SNP Variation Suite (SVS) environment (Golden Helix, Version 7.7.6).

### Preparation of files for analysis

Quality control (QC) was carried out on genotypes imported into the SVS environment. SNPs were removed from analysis if they had a call rate of less than 0.80 or a minor allele frequency of less than 0.05. Cattle were removed from analysis if they had a call rate of less than 0.95. Following QC, 2,008 cattle and 44,338 markers remained for analysis. LD pruning was carried out at r^2^ threshold of 0.5 and 7,841 markers were discarded following pruning^37^. The remaining 36,496 SNPs that passed all QC measures were acceptable for further analysis (Supplementary Table S3).

The collated phenotypic data were merged with the genotype data, creating a dataset containing 1,822 cattle eligible for analysis. A genomic kinship matrix was computed from the population, which was included as a covariate in the GWAS in order to account for relatedness. From this dataset, cattle from five beef breeds were analysed (n = 1492). The breeds included in the analysis were AA, BB, CH, LM and SI (n = 102, 177, 387, 537 and 289, respectively).

### Genome-wide association studies

GWAS were carried out in the SVS environment of Golden Helix using a mixed linear model method, EMMAX^38^, for each breed individually. GWAS resulted in the generation of summary statistics for each trait of interest, i.e. RFI, ADG and FI, for each breed (AA, BB, CH, LI, and SI).

### Meta-Analysis

Following initial breed specific GWAS, meta-analyses were carried out for each trait using the software package METAL^39^. METAL combines *P*-value and direction of effect from each GWAS to conduct Z-score method meta-analysis. METAL analysis results in two outputs, the Z-score for each SNP and a *P*-value for each SNP. A large positive Z-score results in a small *P*-value providing evidence that the allele positively associated with the trait under test. Conversely, a large negative Z-score results in a small *P*-value, showing an allele is negatively associated with the trait^39^. A *P*-value of less than 5 × 10^−5^ was used to denote genome-wide significance as per recent GWAS studies^22^.

### Validation of internationally identified RFI SNPs in Irish beef cattle

The inclusion of internationally identified RFI SNPs (n = 102, Supplementary Table S1) in the current study enabled investigation of their role as markers for feed efficiency in an Irish population of beef cattle.

### Functional annotation of genes

Functional annotation of candidate genes was carried out to gain insight into the underlying biology of RFI, ADG and FI. Database for Annotation, Visualisation and Integrated Discovery (DAVID, version 6.8)^40^ was used for functional annotation. From the meta-analysis, a list of candidate genes was generated using Ensembl’s Variant Effect Predictor. The list contained the nearest gene within a 500 kb window to each nominally significant SNP (*P* < 0.05). DAVID assigned genes to pathways as per the Kyoto Encyclopaedia of Genes and Genomes (KEGG), and determined enrichment of pathways using Fisher’s exact test^41^. In order to account for multiple testing, a Benjamini-Hochberg correction was applied^42^. Pathways were deemed to be significant if they obtained a corrected *P-*value of <0.05. Pathways specifically addressing human diseases and disorders were not included in further analysis of DAVID identified pathways, as these were not relevant to this study.

### eQTL analysis

Samples for eQTL analysis were obtained from CH and Holstein-Friesian cattle that had been genotyped as part of the current study and for which RNA-Seq data were available within our group. The RFI range of the CH and Holstein-Friesian cattle were 1.48 to −0.98 and 1.48 to −1.41 respectively. RNA-Seq raw read counts were collated from liver and muscle tissue analyses carried out by our group in published studies^29,43^ and studies in preparation (Higgins *et al*., McKenna *et al*.) related to feed efficiency traits. Forty-two liver samples and 39 muscle samples were brought forward to eQTL analysis. For eQTL identification, liver and muscle samples were analysed separately.

Raw read counts were filtered and genes with more than 10 instances of zero expression were removed from analysis, resulting in 14,588 and 14,309 genes with expression in the liver and muscle, respectively, remaining for eQTL analysis. Filtered raw read counts were normalised using DESeq2′s *variance stabilizing transformation* (VST) command^44^. Covariates included in DESeq2 were batch, RFI status (i.e. high or low RFI) and breed. VST normalised counts were brought forward for eQTL analysis.

eQTL analysis was carried out using the R package Matrix eQTL^45^. Only SNPs that reached genome-wide significance after meta-analysis (n = 24) and their nearest gene were considered for eQTL analysis. If a nearest gene was greater than 100 kilobases away from a SNP, this combination was not included in eQTL analysis. As part of eQTL analysis RFI, breed and sex were included as covariates. SNPs with a MAF of less than 0.1 or with known functions, i.e. missense mutations, were excluded, as were genes that were not expressed in either tissue of interest, i.e. liver or muscle. This resulted in 11 SNPs in the analysis. A Bonferroni correction was applied to account for the 11 SNPs. If an eQTL reached a *P*-value of 0.0045 or less, it was considered significant after multiple test correction.

## Results

### GWAS and meta-analysis for RFI

**Figure 1:**
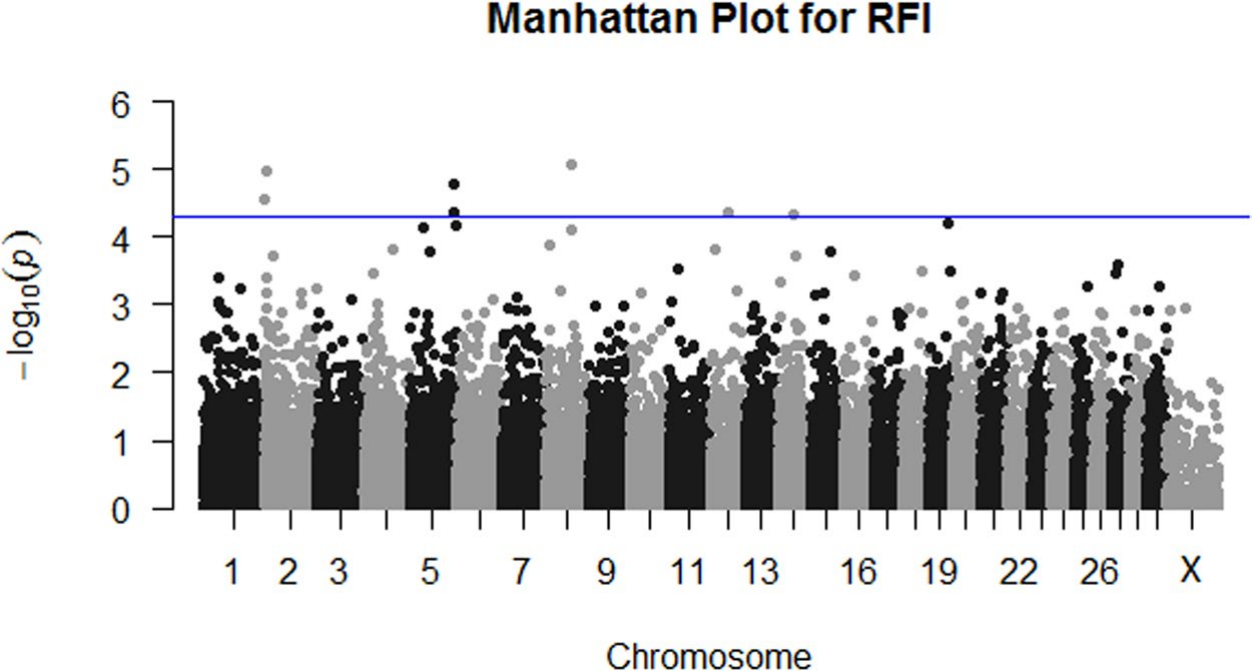
Manhattan plot represents meta-analysis results for RFI, which combined GWAS carried out for five cohorts of Irish beef cattle. The blue line indicates *P*-value < 5 × 10^−5^.

GWAS results generated for RFI by meta-analysis are plotted in Fig. 1. Seven SNPs achieved genome-wide significance for RFI (Table 1). The SNP most associated with RFI was rs43555985, located at chromosome 8 position 69,658,202, a non-coding region 53.4 kb upstream from *GFRA2*. Two variants were within the start-stop coordinates of genes; intronic variant rs110418027 in *SMC1B* and 3′ untranslated region (UTR) variant rs43691372 in *DIS3*. The remaining four SNPs are located in the non-coding region of the Bovine genome and their distance to nearest gene is specified in Table 1. The per breed GWAS results for each of these RFI associated variants are illustrated in Table 2 and Supplementary Table S4. Functional annotation of genes containing or near to nominally significant SNPs for RFI using DAVID did not identify any enriched pathways that survived Benjamini-Hochberg correction^42^.

**Table 1:** SNPs which reached significance (*P* < 5 × 10^−5^) in a multi-breed population of beef cattle after meta-analysis of GWAS results for each respective trait.

SNP I.D.	Trait of interest	Chr_mb	Zscore	*P*-value	Nearby gene	SNP location relative to gene
rs43555985	RFI	8_69	−4.458	8.28E-06	*GFRA2*	53.4 kb upstream
rs41638273	RFI	2_6	4.4	1.08E-05	*SLC40A1*	15.7 kb upstream
rs109695205	RFI	5_113	4.313	1.61E-05	*NFAM1*	26.5 kb upstream
rs110161277	RFI	2_2	4.192	2.76E-05	*PLEKHB2*	143.8 kb downstream
rs110418027	RFI	5_116	−4.089	4.34E-05	*SMC1B*	Intron variant
rs43691372	RFI	12_47	4.082	4.47E-05	*DIS3*	3′ UTR variant
rs42820242	RFI	14_44	4.081	4.48E-05	*IL7*	104.2 kb downstream
rs386023985	ADG	19_48	−6.593	4.32E-11	*ERN1*	7.9 kb upstream
rs135897656	ADG	3_119	6.195	5.83E-10	*CSF2RA*	Intron variant
rs136457441	ADG	19_28	5.936	2.93E-09	*RPL26*	Missense variant
rs110660154	ADG	1_19	5.314	1.08E-07	*SPATA16*	265 kb downstream
rs110780286	ADG	18_15	4.492	7.06E-06	*ITFG1*	Intron variant
rs382426807	ADG	19_43	4.473	7.70E-06	*STAT5A*	Synonymous variant
rs41595251	ADG	9_91	−4.375	1.22E-05	*OPRM1*	269 kb upstream
rs110590483	ADG	11_39	−4.243	2.21E-05	*CCDC85A*	509 kb downstream
rs109252082	ADG	19_53	4.124	3.72E-05	*TBC1D16*	Intron variant
rs41592667	ADG	9_35	4.12	3.78E-05	*FRK*	88 kb downstream
rs41630180	ADG	17_1	−4.097	4.18E-05	*TLL1*	Intron variant
rs41614223	ADG	9_27	−4.086	4.39E-05	*NKAIN2*	8.8 kb downstream
rs137576435	ADG	19_12	−4.079	4.52E-05	*BCAS3*	Intron variant
rs136789347	ADG	23_52	−4.069	4.72E-05	*OR5M10*	13.8 kb upstream
IDBV32000008978	FI	20_67	4.355	1.33E-05	*ADAMTS16*	Synonymous variant
rs55617218	FI	19_14	−4.205	2.61E-05	*HNF1B*	Intron variant
rs109691080	FI	1_6	4.084	4.43E-05	*MAP3K7CL*	58.9 kb upstream

SNP: Single nucleotide polymorphism; RFI: Residual feed intake; ADG: Average daily gain; FI: Feed intake; Chr_mb: Chromosome_megabase.

**Table 2:** Individual breed GWAS results for all genetic variants that reached genome-wide significance following meta-analysis.

Trait	SNP ID	Meta-analysis *P*-value	Direction of Effect	AA *P*-value	BB *P*-value	CH *P*-value	LM *P*-value	SI *P*-value
RFI	rs43555985	8.28E-06	−−−−−	2.14E-01	1.18E-01	5.30E-01	1.24E-03	2.39E-03
	rs41638273	1.08E-05	+++++	1.65E-01	3.16E-03	5.79E-03	9.03E-02	1.74E-01
	rs109695205	1.61E-05	+++++	3.02E-02	4.12E-02	1.09E-01	4.04E-02	2.35E-02
	rs110161277	2.76E-05	+++++	1.68E-01	5.24E-03	1.41E-02	1.50E-01	8.58E-02
	rs110418027	4.34E-05	−−−−−	7.77E-02	8.29E-02	4.74E-02	3.92E-02	7.50E-02
	rs43691372	4.47E-05	+++++	1.74E-01	5.36E-02	6.36E-01	8.67E-03	4.65E-03
	rs42820242	4.48E-05	+++++	1.49E-01	2.74E-01	7.48E-02	1.06E-02	4.41E-02
ADG	rs386023985	4.32E-11	−−−−−	3.43E-01	3.82E-01	4.71E-04	1.16E-07	1.30E-03
	rs135897656	5.83E-10	+++++	1.54E-01	1.70E-01	3.64E-07	1.19E-06	3.01E-01
	rs136457441	2.93E-09	+−+++	4.58E-01	−9.64E-01	4.55E-06	1.88E-05	1.03E-02
	rs110660154	1.08E-07	+++++	3.69E-02	−3.56E-01	5.20E-05	9.81E-02	5.63E-03
	rs110780286	7.06E-06	+++++	2.13E-01	−9.79E-03	3.94E-01	9.86E-02	4.21E-04
	rs382426807	7.70E-06	+−+−+	5.84E-01	−7.69E-01	1.50E-07	5.65E-06	6.85E-01
	rs41595251	1.22E-05	−−−−−−	1.83E-01	−9.94E-01	1.17E-01	4.61E-05	1.70E-02
	rs110590483	2.21E-05	−−−−−	3.60E-01	1.69E-01	5.64E-03	5.12E-01	2.27E-03
	rs109252082	3.72E-05	+++++	8.27E-02	5.49E-01	1.26E-03	5.09E-01	1.07E-02
	rs41592667	3.78E-05	+++++	4.92E-01	−6.02E-01	7.18E-03	8.14E-02	6.75E-03
	rs41630180	4.18E-05	−−−−−	8.08E-01	−1.87E-03	1.25E-01	6.19E-04	2.61E-01
	rs41614223	4.39E-05	−−−−−	2.94E−01	−7.84E-03	9.24E-04	6.27E-02	5.16E-01
	rs137576435	4.52E-05	−−−−−−	3.95E-01	4.11E-01	2.03E-02	7.64E-01	1.66E-04
	rs136789347	4.72E-05	−−−−−−	1.99E-01	6.66E-01	1.35E-01	1.77E-04	5.03E-02
FI	IDBV32000008978	1.33E-05	++++−	6.00E−03	1.69E-03	7.68E-03	3.35E-02	8.59E-01
	rs55617218	2.61E-05	+−−−−	4.22E-01	1.75E-01	2.83E-01	1.92E-04	8.14E-03
	rs109691080	4.43E-05	−++++	6.89E-01	4.45E-01	1.41E-04	1.88E-01	6.53E-03

SNP: single nucleotide polymorphism; RFI: residual feed intake; ADG: average daily gain; Direction of effect: direction of effect of the Illumina A allele; FI: feed intake; AA: Aberdeen Angus; BB: Belgian Blue; CH: Charolais; LM: Limousin; SI: Simmental.

### GWAS and meta-analysis for ADG

**Figure 2:**
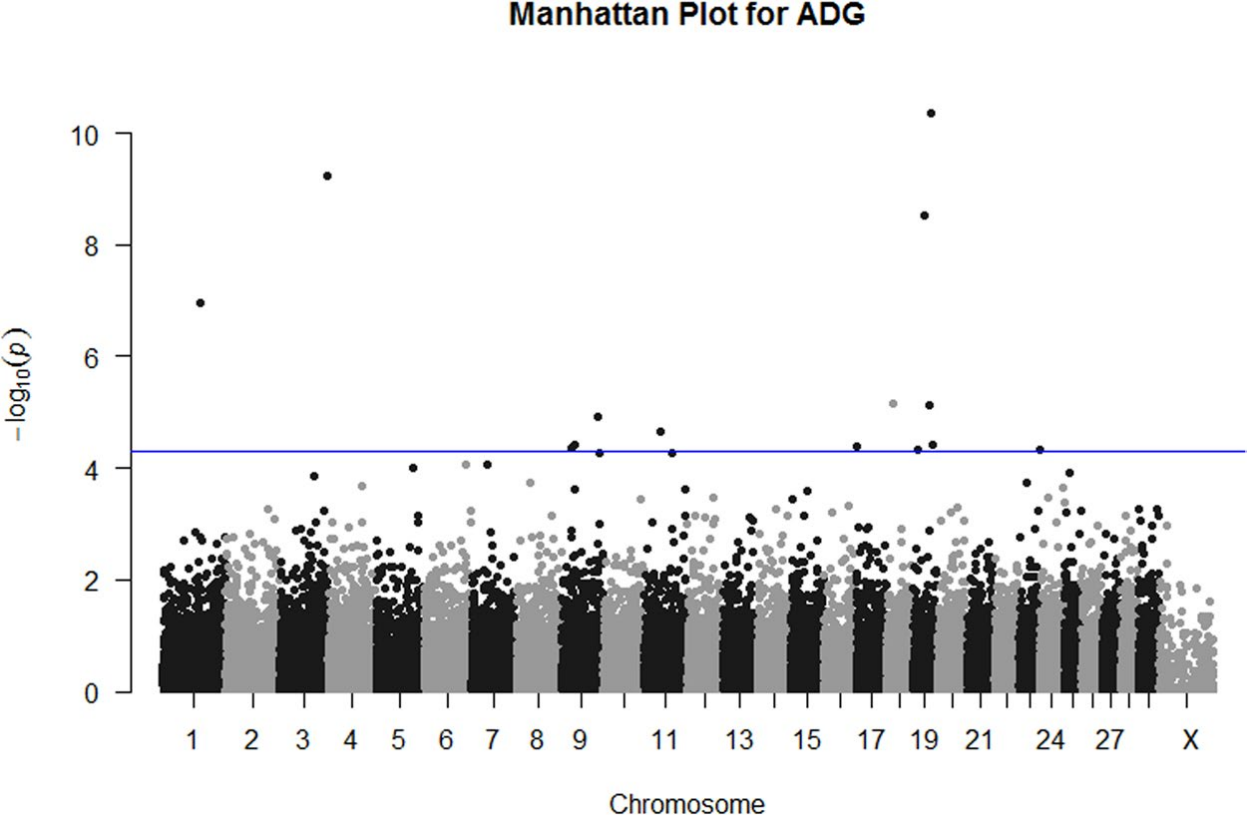
Manhattan plot of meta-analysis results for ADG. Meta-analysis was carried out on GWAS results generated for five breeds of Irish beef cattle. The blue line indicates *P*-value < 5 × 10^−5^.

GWAS results for ADG are illustrated in Fig. 2. A total of 14 SNPs reached genome-wide significance for ADG. The most significantly associated SNP was rs386023985 which is located at chromosome 19 position 48,916,589, 7.9 kb upstream from the *ERN1* gene. One missense variant, rs136457441 in *RPL26*, was associated with ADG. One associated variant was synonymous, rs382426807 in *STAT5A*. Five intronic variants were associated with ADG in the genes: *CSFRA2*, *ITFG1*, *TBC1D16*, *TLL1* and *BCAS3*. The remaining 5 associated SNPs were located upstream or downstream of genes as indicated in Table 1. Individual breed GWAS results for the SNPs associated with ADG following meta-analysis are outlined in Table 2 and Supplementary Table S4.

Functional annotation of genes nearest to nominally significant SNPs for ADG identified 7 pathways that were significantly enriched following Benjamini-Hochberg correction^42^ (Table 3). The thyroid hormone signalling pathway was the most enriched pathway (corrected *P* = 0.01).

**Table 3:** Significant KEGG pathways identified for each trait in a multi-breed population of beef cattle following meta-analysis of GWAS results.

Trait	Biological Process	B-H *P*-value	Number of genes
ADG	Thyroid hormone signalling pathway	0.010	20
ADG	cGMP-PKG signalling pathway	0.011	25
ADG	Vascular smooth muscle contraction	0.015	18
ADG	Retrograde endocannabinoid signalling pathway	0.013	18
ADG	Focal adhesion	0.027	27
ADG	cAMP signalling pathway	0.026	26
ADG	Adherens junction	0.029	13
FI	Axon guidance	0.001	23
FI	Thyroid hormone signalling pathway	0.015	19

B-H *P*-value: Benjamini-Hochberg corrected *P*-value; ADG: average daily gain; FI: feed intake. Pathways were designated as significant if they reached Benjamini-Hochberg corrected *P* < 0.05.

### GWAS and meta-analysis for FI

**Figure 3:**
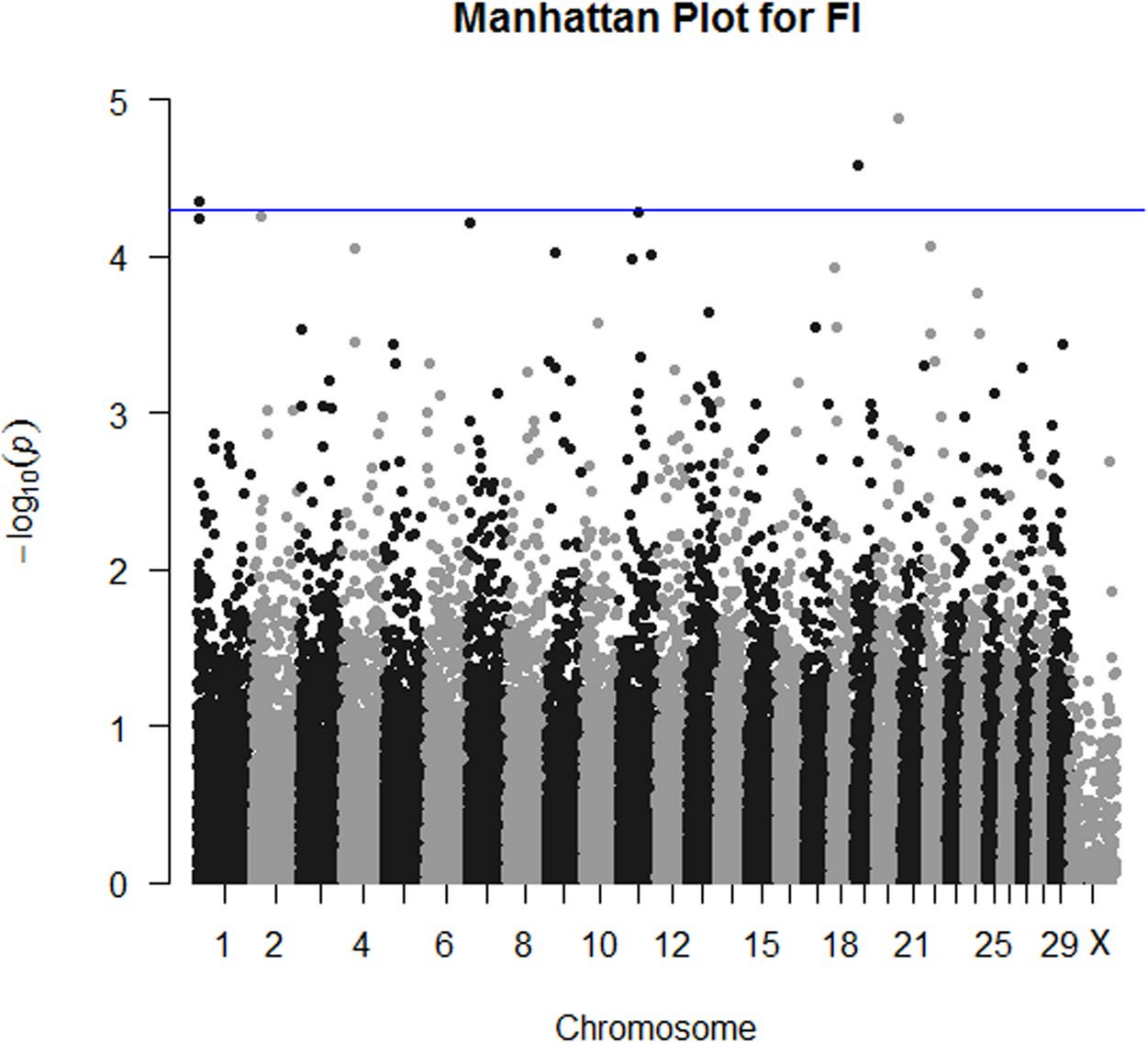
Manhattan plot of FI meta-analysis of GWAS results for Irish beef cattle. The blue line indicates *P*-value < 5 × 10^−5^.

GWAS results for FI are plotted in Fig. 3. Three SNPs reached genome-wide significance for FI (Table 1). Individual breed GWAS results for these variants are presented in Table 2 and Supplementary Table S2. The SNP most associated with FI was IDBV32000008978 located at chromosome 20 position 67,944,737. This is a synonymous variant in *ADAMTS16*. The other two were an intronic variant in *HNF1B* and a variant located 58.9 kb upstream of MAP3K7CL. Functional annotation of the FI SNP results identified two pathways that were significantly enriched after correction; axon guidance and the thyroid hormone signalling pathway (Table 3).

### Validation of internationally identified SNPs in an Irish cattle population

Of the 102 internationally identified RFI SNPs included on the custom IDBv3 genotyping chip, 71 passed all QC measures and were included in the GWAS for RFI in the current study. Two of these SNPs, rs29014641 and rs109500421, were nominally significant in our study but did not survive multiple test correction for this subset of SNPs. This subset of SNPs was not exhaustive for RFI and did not include all variants within quantitative trait loci (QTLs) as identified by Nkrumah, *et al*.^18^, for example. However, a *post-hoc* search for genotyped SNPs within those regions which found that no genetic variants within these QTLs reached genome-wide significance following meta-analysis.

### eQTL analysis of SNPs identified as significant from meta-analysis

**Figure 4:**
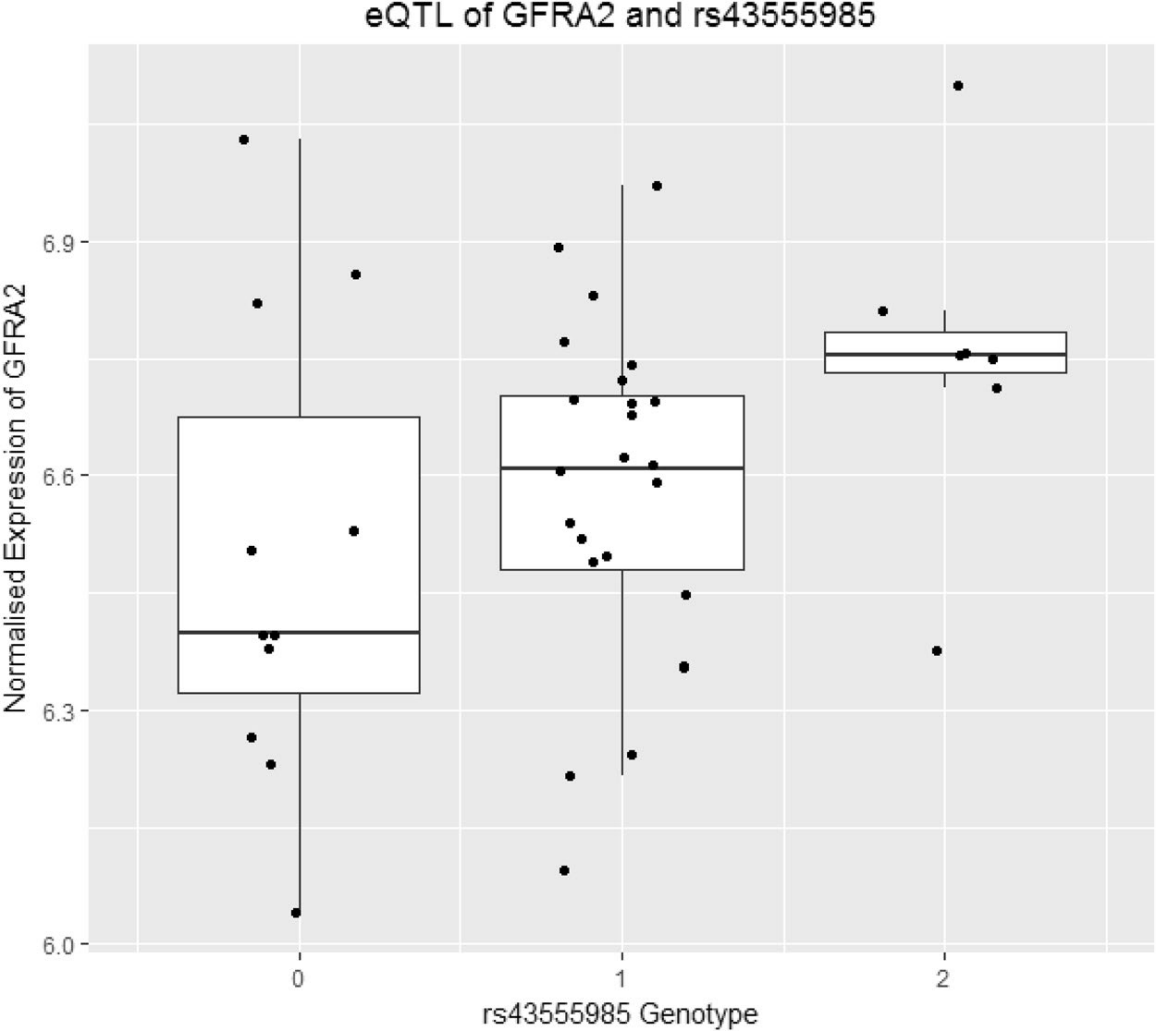
Boxplot representing the relationship between rs43555985 genotypes and normalised liver gene-expression of *GFRA2*. Presence of the minor allele of rs43555985 is correlated with increased expression of *GFRA2*. 0: GG; 1: GA; 2: AA.

Table 4 contains results of eQTL analysis. One cis-eQTL was detected in liver, between rs43555985, the top associated SNP from the RFI GWAS, and *GFRA2* (*P* = 0.0038; survives multiple test correction). eQTL analysis indicated that the minor allele of rs4355985 is associated with increased expression of *GFRA2* (Fig. 4). The same minor allele is associated with lower RFI in the GWAS. The effect of *GFRA2* expression on RFI is presented in Supplementary Fig. S5 on a per genotype basis.

**Table 4:** Results from eQTL analysis of genome-wide significant SNPs in liver and muscle.

SNP	Nearest Gene	Trait	Liver *P*-value	Muscle *P*-value
rs43555985	*GFRA2*	RFI	**0**.0038*	0.25
rs109695205	*NFAM1*	RFI	0.95	0.55
rs110418027	*SMC1B*	RFI	0.15	Not expressed
rs43691372	*DIS3*	RFI	0.96	0.16
rs386023985	*ERN1*	ADG	0.99	0.23
rs110780286	*ITFG1*	ADG	0.83	0.23
rs382426807	*STAT5A*	ADG	0.94	0.72
rs41592667	*FRK*	ADG	0.22	0.19
rs41630180	*TLL1*	ADG	Not expressed	0.33
rs137576435	*BCAS3*	ADG	0.25	0.93
rs109691080	*MAP3K7CL*	FI	Not expressed	0.80

SNP: Single nucleotide polymorphism; ADG: average daily gain; FI: feed intake; RFI: residual feed intake, *eQTLs were designated as significant if they reached *P* < 0.0045.

## Discussion

Despite the economic and environmental benefits of RFI, the trait or indeed any measure of feed efficiency, is not widely adopted within breeding programmes for beef cattle due to the difficulty and expense associated with measuring feed intake^10^. The identification of robust genetic markers of RFI applicable to several breeds, as well as crossbred cattle, would enable the traits inclusion in genomic breeding programmes. This study sought to identify SNPs associated with RFI that could be applicable to Irish beef production enterprises as well as uncovering novel markers of potential use to international beef producers. To unravel the underlying biology causing phenotypic variation in feed efficiency related traits, we carried out eQTL analysis of GWAS-identified variants to study their effect on local gene expression.

rs43555985 was associated with RFI and is an eQTL of the *GFRA2* gene in liver tissue. The minor allele of this SNP was associated lower RFI within each of the individual breed GWAS and following meta-analysis. The minor allele of rs43555985 was also associated with increased expression of *GFRA2* following eQTL analysis. *GFRA2* is a cell-surface receptor that facilitates binding of a member of the glial cell-derived neurotrophic factor family. *GFRA2* knock-out mice are unable to digest food correctly, have impaired salivary secretion and gut motility and exhibit a slower growth rate than wild-type mice while having an increased basal metabolic rate^46^. If increased *GFRA2* expression is associated with improved feed efficiency, the mechanism may involve lowering metabolic rates. Increased metabolic rate leads to increased energy requirements to carry out biological processes and to maintain physiological homeostasis, resulting in less consumed energy being used for growth^47^. It has been illustrated previously that high-RFI lambs have a higher basal metabolic rate than their low-RFI (more desirable) counterparts and low-RFI heifers exhibited lower metabolic rates than their high-RFI (inefficient) counterparts^48,49^. Further investigation and validation of rs43555985 prior to inclusion in genomic breeding programmes is required. Furthermore, rs43555985 is also located 79.9 kb upstream from *XPO7*. *Post-hoc* eQTL analysis for this gene illustrated that there is no statistically significant relationship between rs43555985 genotype and *XPO7* expression.

The second most statistically significant SNP for RFI, rs41638273, maps to a region of chromosome 2 that contains the *SLC40A1* gene. This region is also the site of a QTL for RFI which contains the myostatin gene^50^. Specific mutations in the myostatin gene have been linked with increased muscle growth traits^51^. Improved feed efficiency was associated with double muscled Angus steers by Cafe *et al*.^52^ when compared to lesser muscled counterparts^52^. *DIS3*, a gene nearby to a variant associated with RFI in the current study, encodes a protein involved in RNA metabolism^53^ and has been linked with feed conversion efficiency in pigs^54^.

The minor allele of rs386023985 was negatively associated with ADG following GWAS meta-analysis in the current study. Similarly, this variant was negatively associated with ADG within each individual breed GWAS conducted, this SNP also reached genome-wide significance within the LM individual breed GWAS. rs386023985 has not previously been associated with ADG or other growth traits in cattle. The gene nearest to rs386023985 is *ERN1 (IRE1)*, a sensor of metabolic stress, is involved in the unfolded protein response^55^.

Copy number variation in *RPL26*, a ribosomal protein gene, has been linked to RFI divergence in Holstein cows^56^. A variant identified in this study, rs136457441, is a missense variant in *RPL26* causing an isoleucine to threonine change at amino acid position 67 in the RPL26 protein. This variant, associated with ADG following meta-analysis in the current study and reached genome-wide significance for ADG within the CH individual breed GWAS, is located in exon 3 of *RPL26*. rs136457441 has been designated as tolerated by the Sorting Tolerant from Intolerant (SIFT) algorithm, which predicts whether amino acid substitutions effect protein function^57^. Further investigation into the functional effect of this mutation is required to elucidate its biological role in ADG.

An exonic variant associated with ADG is rs382426807, a synonymous variant in *STAT5A*. This gene encodes a transcription factor that can be activated as part of the somatotropic axis, which is the pathway involved in the secretion of growth hormone and skeletal muscle growth^58^. *STAT5A* has been associated with increased live weight gain in Polish Black-and-White bulls^59^ and increased expression of the growth hormone receptor, an upstream activator of STAT5A, has been previously demonstrated in efficient beef heifers by Kelly *et al*.^60^.

Five variants associated with ADG were located in introns of the following genes: *TLL1*, *CSF2RA*, *ITFG1*, *TBC1D16* and *BCAS3*. *TLL1* encodes a member of the tolloid family metalloproteases that have been previously implicated in the cleavage and development of myostatin in humans^61^. Myostatin in its normal state negatively regulates muscle growth. The production of aberrant myostatin protein isoforms results in the development of the double muscle phenotype^51^.

*CSF2RA* encodes for a granulocyte/macrophage colony stimulating factor^62^. *ITFG1*, the gene within which rs110780286 is located, is involved in T-cell differentiation and may induce the production of anti-inflammatory cytokines^63^. It has been previously illustrated that immune genes and immune pathways are associated with variation in feed efficiency and ADG in cattle^64,65^. Several groups have suggested that the immune system plays a key role in weight gain and feed efficiency in cattle. For example, Reynolds *et al*.^64^ found that steers with higher ADG have lower immunity related gene expression^64^ and it has been demonstrated that cattle with poor feed efficiency had increased activation of their immune system^65^. It is possible that cattle with poor feed efficiency and low ADG are experiencing chronic inflammation which results in poor feed efficiency which has been suggested previously by Alexandre *et al*.^65^ following analysis of beef cattle divergent in RFI and by Mani *et al*.^66^ upon investigation of inflammation in RFI divergent pigs^65,66^.

rs41595251 is associated with ADG and is a variant located upstream from *OPRM1*, the µ-opioid receptor gene, on chromosome 9. *OPRM1* has been associated with increased food intake in humans^67^. ADG-associated SNP rs136789347 is nearby to *OR5M10* which encodes for an olfactory receptor in humans^68^. Olfactory receptors have been suggested as one method by which the endocannabinoid system stimulates the feeding drive in mice^69^. rs41614223 is located downstream from the transcriptional start site of *NKAIN2*, which produces a Sodium-Potassium ATPase involved in action potential generation in neurons^70^. Each of these genes, *OPRM1*, *OR5M10* and *NKAIN2* have a neurological function. There is evidence from bovine studies^71,72^ that there is significant neurological control of food consumption. The association of these neurological genes with ADG in this cohort of beef cattle may further indicate that feeding behaviour in cattle may also be subject to some degree of neurological control^73^. Further investigation is required to investigate the role neurological systems play in modulating the development of divergent RFI and related traits in cattle.

rs41592667 is upstream of *FRK* which encodes for tyrosine-protein kinase FRK. Gene sets enriched for cell cycle-related genes, similar to *FRK*, have previously been shown to be associated with feed intake and feed efficiency in beef cattle^74^. *TBC1D16*, a gene which encodes for a GTPase and contains the intronic variant rs109252082, has been associated with growth rate in pigs^75^. Despite these SNPs not being associated with feed efficiency or component traits prior to the current study, they are near to, or within, genes that have been associated with feed efficiency related traits previously.

*HNF1B*, nearby to a variant associated with FI, has previously been identified as differentially expressed in Holstein cattle divergent for RFI^76^, and this gene is a target of miR-802, which has been identified as upregulated in high RFI cattle^77^. The silencing of *HNF1B* in mice leads to impaired insulin sensitivity^78^. However, previous work by our group has shown that RFI divergent beef cattle have similar levels of insulin sensitivity and it is unlikely that insulin sensitivity plays a role in RFI divergence^28^. Further work is required to understand the contribution of *HNF1B* to the development of divergence in FI. In this study the variant IDBV332000008978 was associated with FI. This variant is a synonymous variant within the *ADAMTS16* gene which is a member of ADAMTS protease family and has previously been identified as associated with FCR in pigs^79^.

Following functional gene set enrichment analysis using DAVID, the thyroid hormone signalling pathway was found to be most enriched for ADG. Thyroid hormones play a key role in the regulation of basal metabolism in mammals^80^, although, it has been demonstrated previously that the levels of thyroid hormones are not related to RFI status in heifers^81^. However, in a study of dairy cattle is was reported that low levels of thyroid hormones are associated with lower RFI^82^. Further investigation into the role of the thyroid hormone signalling pathway is warranted to further elucidate the role this biological mechanism plays in the divergence of RFI in cattle. The retrograde endocannabinoid signalling pathway was also found to reach significance level in the list of nominal significant genes for ADG. It has been demonstrated that the endocannabinoid system plays a role in inducing food intake and modulating energy expenditure and feed intake in mice^83,84^. It is possible that alterations in genes in the retrograde endocannabinoid pathway may also stimulate or inhibit feeding behaviours in cattle which may impact on feed efficiency. It has been observed previously that low RFI cattle have fewer daily feeding events and have a lower eating rate than high RFI cattle^85^. Focal adhesion was another pathway found to be enriched for nominally significant ADG associated SNPs. Focal adhesion is a pathway involved in cell motility, proliferation and survival. This pathway is dependent upon focal adhesion kinase^86^. *PTK2*, the gene encoding for focal adhesion kinase has been previously noted as downregulated in high-RFI animals from a population of dairy cattle^87^.

## Conclusion

In this study we illustrate genome-wide associations between SNPs and RFI and its component traits in beef cattle. In total, we identified 24 SNPs as reaching statistical significance for RFI, ADG and FI in a multi-breed cohort of beef cattle. Several of the SNPs identified in this study are located nearby or within genes related to immune function, muscle growth and development, and neurological pathways. The identification of a novel eQTL for RFI at *GFRA2* also represents an insight into the biology of feed efficiency.

Due to the small sample size of our individual breed GWAS, which we used meta-analysis to overcome, all identified SNPs and the eQTL must be validated, both in larger Irish and international populations before incorporation into genomic assisted beef cattle breeding programmes. Furthermore, validation is required in larger reference populations to account for the LD and genetic heterogeneity which exists between breeds of cattle.

An additional method which may have been employed to increase sample size could have been single step GWAS (ssGWAS)^88–90^. ssGWAS incorporates genotypes, phenotypes and pedigree information to calculate genomic estimated breeding values for animals with or without genotypes^88^.

It is important to ensure that the SNPs influence these traits and have no negative impact on other economically important production traits. SNPs with a validated desirable effect can be included in Irish and international genomic assisted breeding programmes to facilitate the rapid and cost effective selection of more feed efficient beef cattle.

## Electronic supplementary material

Supplementary Table S1

Supplementary Table S2

Supplementary Table S3

Supplementary Table S4

Supplementary Figure S5
