# Supplementary material for: GWAS and eQTL analysis identifies a SNP associated with both residual feed intake and GFRA2 expression in beef cattle
Source: Sci Rep. 2018 Sep 24;8:14301. doi: 10.1038/s41598-018-32374-6 (PMC6155370; doi:10.1038/s41598-018-32374-6)
Supplement: Supplementary file 5 — Supplementary Figure S5 [file 41598_2018_32374_MOESM5_ESM.docx]

Supplementary Figure S5. The relationship between *GFRA2* expression and RFI for genotypes of rs43555985.

Supplementary Figure S5a.

Supplementary Figure S5b.

Supplementary Figure S5c.

Supplementary Figure S5d.

Supplementary Figure S5. Scatterplots illustrating the relationship between *GFRA2* expression and RFI for genotypes at rs43555985.

Supplementary Figure S5a illustrates the relationship between *GFRA2* expression and RFI for all genotypes of rs43555985. Supplementary Figure S5b illustrates the relationship between *GFRA2* expression and RFI for animals with major allele homozygous genotypes at rs43555985. Supplementary Figure S5c highlights the relationship between *GFRA2* expression and RFI for animals with heterozygous genotypes at rs43555985. Supplementary Figure S5d illustrates the relationship between *GFRA2* expression and RFI for animals with minor allele homozygous genotypes at rs43555985.
